# Patient-Reported Outcomes After Adult Spinal Deformity Surgery: Are There Differences Between Primary and Revision Surgery?

**DOI:** 10.7759/cureus.83793

**Published:** 2025-05-09

**Authors:** Amalie Schramm, Martin Heegaard, Lærke C Ragborg, Rosemarie E Høi-Hansen, Lars V Hansen, Benny Dahl, Martin Gehrchen, Søren Ohrt-Nissen

**Affiliations:** 1 Spine Unit, Department of Orthopaedic Surgery, Rigshospitalet, Copenhagen, DNK

**Keywords:** adult spinal deformity, asd revision surgery, health-related quality of life, patient satisfaction, spine deformity surgery

## Abstract

Background: Surgical treatment of adult spinal deformity (ASD) can significantly improve health-related quality of life (HRQoL) but is associated with a high revision rate. Whether the same patient satisfaction can be expected after revision surgery compared to primary surgery is uncertain.

Purpose: This study aimed to compare patient satisfaction between revision and primary ASD surgery.

Methods: We conducted a retrospective study on patients with ASD undergoing primary or revision surgery on ≥ five levels. We included adult patients from a single center between 2010 and 2020 who had completed ≥two-year-postoperative HRQoL questionnaires. Patients were divided into two groups: primary surgery and revision surgery. Health-related quality of life and treatment satisfaction were assessed with the Scoliosis Research Society Patient Outcome Questionnaire (SRS-22r) and the European Quality of Life (EQ-5D-3L) questionnaires and compared between groups.

Results: A total of 185 patients completed the postoperative questionnaires (97 primary surgery; 88 revision surgery). The mean age was 59.8±16.1 years in the revision group and 50.7±20.4 in the primary group (p<0.001). The median satisfaction score was 4.0 (3.0-4.5) in the revision group and 4.0 (3.5-4.6) in the primary group (p=0.096). The median SRS-22r subscore was 3.0 (2.4-3.6) vs. 3.3 (2.5-3.9) (p=0.087). The EQ-5D-3L index score was 0.37 (0.30-0.53) vs. 0.42 (0.30-0.63) (p=0.106) for the revision surgery group and primary surgery group, respectively.

Conclusion: We found no difference in overall patient-reported HRQoL and treatment satisfaction between revision and primary surgery. Although revision surgery is associated with increased morbidity and risk of complications, patients can expect the same patient satisfaction compared to their primary surgery in the absence of complications.

## Introduction

Adult spinal deformity (ASD) is a complex condition causing pain, disability, and, in some cases, dissatisfaction with appearance. This significantly impacts health-related quality of life (HRQoL) [1], and patients suffering from ASD have HRQoL similar to people suffering from lung cancer, stroke, or heart failure [2,3]. The prevalence of ASD has increased due to an aging population, leading to an increased burden of disease [1]. Thus, understanding how to manage patients with ASD to improve HRQoL is essential.

Surgical treatment for ASD consists of spine realignment using osteotomies, decompression, and fusion [4]. Patients undergoing surgical treatment have shown better HRQoL compared with patients receiving nonsurgical treatment in terms of decreased pain and disability [5-9]. However, ASD surgery is associated with a high risk of complications and revision surgery with complication rates up to 70% and revision rates around 25% within two years of surgery [10-12]. Complications related to ASD surgery and reasons for reoperation include infection, dural tears, bleeding, acute and delayed neurological deficits, proximal junctional failure, and mechanical failure with rod breakage or screw loosening, often due to pseudoarthrosis [13].

Previous studies on HRQoL for patients with ASD undergoing revision surgery have shown somewhat conflicting results. While some studies show significant worsening of HRQoL after revision surgery [11,14,15] others have shown no difference in HRQoL between patients undergoing revision or primary surgery [16-19]. The inconsistent findings from previous studies may be due to short follow-up time and inadequate study design [11,14-17,19].

This study aimed to compare patient satisfaction as well as patient-related outcomes (PRO) on HRQoL for patients with ASD undergoing revision and primary surgery with a minimum of two-year follow-up.

## Materials and methods

Study design

We conducted a retrospective, single-center, cross-sectional study on patients with ASD undergoing primary or revision surgery. We included all adult patients from a tertiary spine unit who had either primary or revision ASD surgery on five or more levels from January 1st, 2010, to December 31st, 2019.

Primary surgery was defined as the first spine realignment surgery with instrumentation on five levels or more.

We included patients who had completed their ≥two-year postoperative HRQoL questionnaires. Patients undergoing surgery during the two-year follow-up period were excluded. The surgery-free follow-up period was chosen to represent a ‘steady state’ for the patient.

Patients were identified through procedure codes in the department’s operation booking system and were divided into two groups: (1) primary surgery (control group) and (2) revision surgery. Health-related quality of life and treatment satisfaction were assessed with questionnaires SRS-22r and EQ-5D-3L, with patient satisfaction chosen as the primary outcome. These questionnaires were collected prospectively as part of the routine two-year postoperative clinical follow-up. An additional email was sent to non-responders. Patient satisfaction was assessed using the SRS-22r subdomain 'treatment satisfaction'. Additional PROs were compared in a cross-sectional design since preoperative data were incomplete. A subgroup analysis of the available preoperative data for both groups was made as well. Patient demographics were collected from patient charts. The Charlson Comorbidity Index Score (CCI Score) was calculated at the time of surgery. Preoperative, postoperative, and two-year control x-ray parameters were measured and calculated using the validated software KEOPS (SMAIO, Lyon, France) [20]. All cases were operated on by a dual-surgeon team of specialized orthopedic spine surgeons. Surgical goals and strategies were agreed upon at a preoperative planning meeting, but certain techniques may differ between surgeons.

X-ray measurements

The sagittal vertical axis (SVA) is the horizontal distance between a plumb line from the center of C7 to the superior-posterior endplate corner of S1, and describes the spine’s sagittal balance, with the normal range being ±5 mm. The pelvic incidence (PI) is an angle between a line perpendicular to the midpoint of the sacral plate and a line from the sacral plate to the center of the midpoint between the femoral heads, and describes the pelvic position in the sagittal plane, with the normal range being 55°±10°.

Statistics

Data were assessed for normal distribution with histograms and reported as means with standard deviations (SD) or medians with interquartile range (IQR). Parametric data were compared using an independent two-tailed samples t-test, and non-parametric data with the Mann-Whitney U test. Statistical significance was defined as a p-value <0.05. All statistical analyses were performed using R v 4.2.2 (R Foundation for Statistical Computing, Vienna).

The study was approved by the Danish Patient Safety Authority (no. R-21054762) and the Danish Data Protection Agency / Knowledge Center for Data Reviews in the Capital Region (no. p-2023-14406).

## Results

In the cross-sectional study on postoperative PRO, we identified 420 patients undergoing ASD surgery between 2010-2020 of which 185 eligible patients were included in the final analysis: 97 in the primary surgery group and 88 in the revision surgery group. A total of 75 out of 97 primary surgery patients (77%) and 36 out of 88 revision surgery patients (41%) had available preoperative questionnaires. Mean age in the revision group was 59.8±16.1 years compared to 50.7±20.4 years in the primary surgery group (p<0.001) (Table 1). Around 72% in the revision surgery group vs. 59% in the primary surgery group were female (p=0.068), and the median CCI Score was 2 (1-3) vs. 2 (0-3) (p=0.017) (Table 1). Median time of follow-up in the revision group was 4.6 years (2.3-7.2) vs. 3.32 years (2.0-7.7) (p=0.585) in the primary surgery group.

**Table 1:** Patient demographics by group Data are mean (± SD), median (IQR) or counts (%). CCI: Charlson Comorbidity Index

	Primary surgery (n = 97)	Revision surgery (n = 88)	P-value
Age (years), mean (SD)	50.7 (20.4)	59.8 (16.1)	<0.001
Sex (female)	57 (59%)	63 (72%)	0.068
CCI score, median (IQR)	2 (0-3)	2 (1-3)	0.017
Follow up-time (years), median (IQR)	3.32 (2.01-7.70)	4.62 (2.30-7.17)	0.585

Mean preoperative sagittal vertical axis (SVA) was 85±75 mm in the revision group and 55±79 mm in the primary surgery group (p = 0.010). Correction of SVA was 34±64 mm vs. 14±63 mm (p=0.037). The mean two-year postoperative pelvic incidence (PI) was 54±16° vs 49±12° (p=0.042). Aside from this, there were no differences in radiographic parameters between the two groups (Table 2).

**Table 2:** Radiographic parameters by group Data are mean (± SD).

	Primary surgery (n = 97)	Revision surgery (n = 88)	P-value
Pelvic incidence (°)			
Preoperative	50 (14)	54 (15)	0.055
2-year postoperative	49 (12)	54 (16)	0.042
Pelvic tilt (°)			
Preoperative	21 (12)	23 (11)	0.169
2-year postoperative	19 (11)	22 (9)	0.079
Sacral slope (°)			
Preoperative	29 (13)	31 (13)	0.381
2-year postoperative	30 (11)	32 (14)	0.415
Global lordosis (°)			
Preoperative	51 (19)	50 (18)	0.874
2-year postoperative	51 (13)	52 (25)	0.694
Global kyphosis (°)			
Preoperative	56 (28)	58 (26)	0.638
2-year postoperative	61 (19)	64 (16)	0.281
SVA (mm)			
Preoperative	55 (79)	85 (75)	0.01
2-year postoperative	45 (60)	61 (55)	0.077
Correction of SVA	14 (63)	34 (64)	0.037

**Figure 1:**
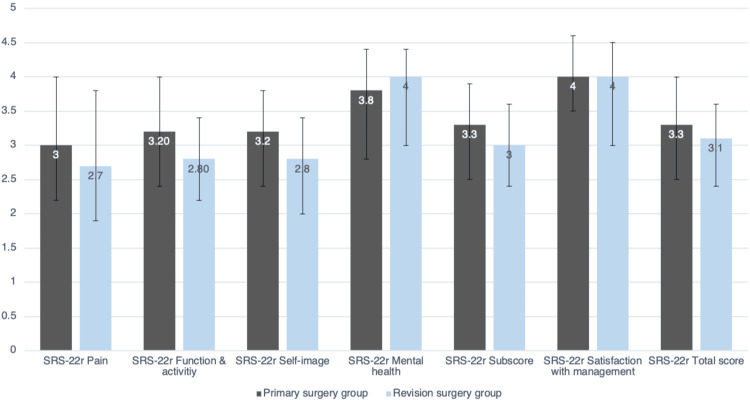
SRS-22r scores in all domains Data are median and IQR. SRS-22r: Scoliosis Research Society Patient Outcome Questionnaire

**Figure 2:**
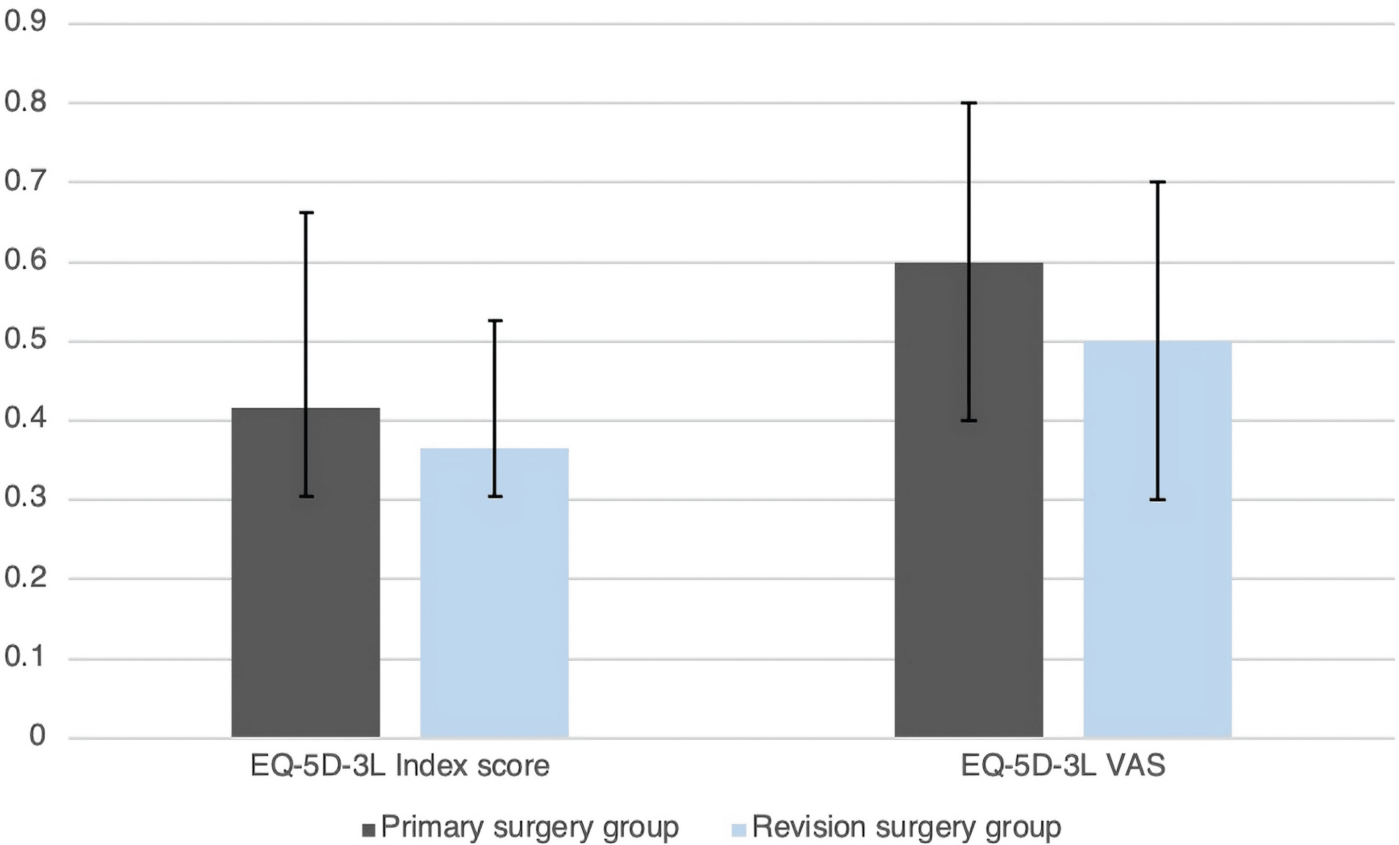
EQ-5D-3L for both domains and groups Data are median and IQR. EQ-5D-3L: European Quality of Life - 5 Dimensions - 3 Levels (patient questionnaire)

The median satisfaction score was 4.0 (3.0-4.5) in the revision group and 4.0 (3.5-4.6) in the primary group (p=0.096). The median SRS-22r subscore was 3.0 (2.4-3.6) vs. 3.3 (2.5-3.9) (p=0.087). The median EQ-5D-3L index score was 0.37 (0.30-0.53) vs. 0.42 (0.30-0.63) (p=0.106) for the revision surgery group and primary surgery group, respectively (Figures 1-2 and Table 3). On the SRS-22r-domains ‘Function and activity’ and ‘Self-image’, we found a statistically significant lower score in the revision group compared to the control group (p=0.012; p=0.025) with a difference between groups in both domains of 0.4 (Table 3).

**Table 3:** Postoperative patient-reported outcome by group Data are median (IQR). SRS-22r: Scoliosis Research Society Patient Outcome Questionnaire, EQ-5D-3L: European Quality of Life - 5 Dimensions - 3 Levels (patient questionnaire)

	Primary surgery (n = 97)	Revision surgery (n = 88)	P-value
SRS-22r Pain	3.0 (2.2-4.0)	2.7 (1.9-3.8)	0.212
SRS-22r Function & activity	3.2 (2.4-4.0)	2.8 (2.2-3.4)	0.012
SRS-22r Self-image	3.2 (2.4-3.8)	2.8 (2.0-3.4)	0.025
SRS-22r Mental health	3.8 (2.8-4.4)	4 (3.0-4.4)	0.705
SRS-22r Sub score	3.3 (2.5-3.9)	3.0 (2.4-3.6)	0.087
SRS-22r Satisfaction with management	4.0 (3.5-4.6)	4.0 (3.0-4.5)	0.096
SRS-22r Total score	3.3 (2.5-4.0)	3.1 (2.4-3.6)	0.830
EQ-5D-3L Index score	0.416 (0.304-0.633)	0.365 (0.304-0.526)	0.106
EQ-5D-3L Self-perceived health/VAS	60% (40-80%)	50% (30-70%)	0.065

We identified 111 patients eligible for subgroup analyses of preoperative data: 75 in the primary group and 36 in the revision group. In the subgroup analysis of the preoperative PRO, we found a statistically significant lower score in the revision group on EQ-5D-3L Index score compared to the primary surgery group (median 0.331 (0.240-0.433) vs 0.429 (0.317-0.589), p = 0.007) (Table 4). There was no significant difference between groups in the remaining domains preoperatively. The subgroup analysis of the postoperative PRO had findings similar to the cross-sectional analyses, with no difference between groups in satisfaction with management, but significant differences in SRS-22r domains ‘Function and activity’ and ‘Self-Image’.

**Table 4:** Subgroup analysis on available pre- and postoperative patient-reported outcome by group Data are median (IQR). SRS-22r: Scoliosis Research Society Patient Outcome Questionnaire, EQ-5D-3L: European Quality of Life - 5 Dimensions - 3 Levels (patient questionnaire)

	Preoperative			Postoperative		
	Primary (n = 75)	Revision (n = 36)	P-value	Primary (n = 75)	Revision (n = 36)	P-value
SRS-22r Pain	2.0 (1.6-3.1)	2.0 (1.6-2.8)	0.820	3.2 (2.2-4.2)	2.8 (2.2-4.0)	0.319
SRS-22r Function & activity	2.8 (2.2-3.7)	2.6 (2.2-3.0)	0.119	3.2 (2.6-4.0)	2.8 (2.2-3.4)	0.012
SRS-22r Self-image	2.2 (1.8-3.0)	2.1 (1.6-3.4)	0.875	3.4 (2.6-4.0)	2.7 (1.8-3.4)	0.010
SRS-22r Mental health	3.6 (2.8-4.2)	3.4 (2.4-4.0)	0.482	3.8 (3.0-4.5)	3.9 (2.6-4.4)	0.813
SRS-22r Sub score	2.7 (2.2-3.4)	2.5 (2.2-3.3)	0.416	3.4 (2.8-4.0)	3.0 (2.3-3.8)	0.055
SRS-22r Satisfaction with management	-	-	-	4.0 (3.5-5.0)	4.0 (2.6-4.9)	0.383
SRS-22r Total score	-	-	-	3.4 (2.8-4.0)	3.1 (2.3-3.9)	0.550
EQ-5D-3L Index score	0.429 (0.317-0.589)	0.331 (0.240-0.433)	0.007	0.430 (0.317-0.633)	0.335 (0.253-0.526)	0.058
EQ-5D-3L Self-perceived health	50% (30-80%)	50% (30-70%)	0.292	60% (40-80%)	50% (20-60%)	0.054

## Discussion

We conducted a retrospective, single-center, cross-sectional study on HRQoL of patients with ASD undergoing revision surgery and primary surgery. We found no overall differences in the postoperative HRQoL between patients with ASD undergoing revision surgery compared to primary surgery overall based on satisfaction with management, SRS-22r subscore, total score, and EQ-5D-3L Index score. A statistically significant lower score in the SRS-22r domains ‘Function and activity’ and ‘Self-image’ was seen in the revision surgery group. The clinical implication of this difference is questionable. Crawford et al. reported the minimal clinical important difference (MCID) for these domains to vary between 0.19 and 1.23 for ‘self-mage’ and 0.23 to 0.60 for ‘Function and Activity’ [21]. The difference in our study is within a difference of 0.4 in both domains.

Our baseline radiological parameters are overall comparable to other studies of patients undergoing primary and revision ASD surgery [14,16,22]. The revision group was older, more comorbid, and had a greater preoperative SVA compared to the primary group. We expected these differences to contribute to a substantially worse PRO in the revision group, but this was not the case. Age, comorbidity, and high SVA are all known risk factors for complications and/or revision surgery [14,23-25]. Higher age is associated with a higher risk of postoperative complications, clinical failure [23], and mechanical failure by proximal junctional kyphosis [25]. High SVA is associated with a higher risk of proximal junctional kyphosis [25], radiological and implant-related complications [24], and revision surgery [14]. Increased comorbidity is associated with readmission [17] and major complications [19].

When assessing our HRQoL results, these are in accordance with previous studies [16-19], showing no difference in HRQoL after a two-year follow-up. Núñez-Pereira et al. [16] and Lee et al. [17] examined the impact of readmission [17] and reoperation [16,17] in patients with ASD on HRQoL and found minor differences between groups at one-year follow-up, but no significant difference at two-year follow-up. Scheer et al. [18] found a lower SRS-22 satisfaction with management score two years postoperatively for patients undergoing revision surgery compared with no revision, but otherwise no difference between groups. Auerbach et al [19] analyzed the impact of minor and major complications on HRQoL in patients with ASD undergoing three-column osteotomy. They found no difference in SRS-22 subscore at the last follow-up between patients experiencing major complications, minor complications, and no complications [19].

Contrary to our study, both Durand et al. [15] and Passias et al. [11,14] found a poorer HRQoL for patients undergoing revision surgery [14,15] and readmission after index surgery [11]. Durand et al. [15] conducted a multicenter study on 1256 patients undergoing ASD surgery. The authors found a decrease in SRS-22 patient satisfaction and total score after revision surgery and a further decline after their second revision surgery, indicating a declining HRQoL with increasing numbers of revision surgeries. Durand et al. [15] and Passias et al. [11,14] did not exclude or adjust for patients developing new revision-requiring symptoms before follow-up. Undeniably, completion of the questionnaires just preoperatively or close to revision surgery could lead to falsely low HRQoL scores. We excluded patients who signed up for revision surgery at follow-up and chose to only include patients with a two-year surgery-free period to highlight findings associated with a “steady-state” condition.

There are several strengths and limitations of this study that should be mentioned. Firstly, we have conducted a single-center study which limits the risk of confounders in the treatment protocol, since all patients have been treated by the same team of surgeons with similar preoperative planning, operative instrumentation, postoperative rehabilitation, and follow-up. Additionally, this follow-up time after revision surgery is one of the longest in the published literature and provides insight into what patients can expect beyond one or two years if they remain free of further revisions. The main limitation of this study is the cross-sectional design with a lack of sufficient baseline information on HRQoL. The main outcome, patient satisfaction, is not affected by the lack of preoperative data, but it would have improved the granularity of the results to have a better understanding of the patients preoperatively. The subgroup analysis indicates that both groups improve their HRQoL after surgery, but with only preoperative data on 41% in the revision group and 77% in the primary surgery group, this cannot be concluded with certainty, though it is also supported by a multitude of studies [21,26-28]. Another limitation of our study is the sample size. Limited by sample size, we were unable to subgroup the revision group according to the number of revisions to assess a potential dose-response relationship or a threshold. Lastly, there is a generally non-significant lower score on the postoperative EQ-5D-3L in both domains for the revision group in the cross-sectional analysis, which could be caused by a type-2 error (Table 3).

During the study period (2010-2020), there was a continuous development in adult spine deformity surgery. These include changes in surgical technique, surgical strategy, as well as implants. A previous paper from our group shows a decrease in revision rates during the study period [29]. However, the indications for revisions were the same, and from a PRO-perspective, we have no indication that patients undergoing revision have changed during the study period.

Our data aids in the understanding of the ASD patient trajectory when the need for revision occurs. Although revision surgery is, for many reasons, an undesirable outcome, it does not indicate a poorer end result of the surgery, provided that additional complications do not occur. Surgeons may use this information to give patients realistic expectations when embarking upon ASD revision.

## Conclusions

In conclusion, we found no differences in patient satisfaction for patients with ASD having revision surgery compared to primary surgery. We found a lower SRS-22r ‘Function and activity’ and ‘Self-image’ score, but the clinical implication of this is unclear. Thus, although revision surgery is associated with higher morbidity and risk of complications, our findings indicate that patients can expect similar PRO more than two years after surgery.
